# *PARP9* affects myocardial function through TGF-**β**/Smad axis and pirfenidone

**DOI:** 10.17305/bb.2024.10246

**Published:** 2024-10-01

**Authors:** Nannan Chen, Lianzhi Zhang, Zhang Zhong, Wenjia Zhang, Qunlin Gong, Nan Xu, Yimeng Zhou, Jiahong Wang, Pengxiang Zheng

**Affiliations:** 1 Department of Cardiology, Yangpu Hospital, School of Medicine, Tongji University, Shanghai, China

**Keywords:** Cardiac arrhythmias, poly (ADP-ribose) polymerase 9 (PARP9), pirfenidone (PFD), TGF-β/Smad signaling pathway, myocardial fibrosis.

## Abstract

Cardiac arrhythmias are often linked to the overactivity of cardiac fibroblasts (CFs). Investigating the impact of poly (ADP-ribose) polymerase 9 (*PARP9*) on Angiotensin II (Ang II)-induced fibroblast activation and the therapeutic effects of pirfenidone (PFD) offers valuable insights into cardiac arrhythmias. This study utilized weighted gene co-expression network analysis (WGCNA), differential gene expression (DEG) analysis, protein–protein interaction (PPI), and receiver operating characteristic (ROC) analysis on the GSE42955 dataset to identify the hub gene with a significant diagnostic value. The ImmuCellAI tool revealed an association between *PARP9* and immune cell infiltration. Our in vitro assessments focused on the influence of PFD on myofibroblast differentiation, transforming growth factor-beta (TGF-β) expression, and Ang II-induced proliferation and migration in CFs. Additionally, we explored the impact on fibrosis markers and the TGF-β/Smad signaling pathway in the context of *PARP9* overexpression. Analysis of the GSE42955 dataset revealed *PARP9* as a central gene with high clinical diagnostic value, linked to seven types of immune cells. The in vitro studies demonstrated that PFD significantly mitigates Ang II-induced CF proliferation, migration, and fibrosis. It also reduces Ang II-induced *PARP9* expression and decreases fibrosis markers, including TGF-β, collagen I, collagen III, and α-SMA. Notably, *PARP9* overexpression can partially counteract PFD’s inhibitory effects on CFs and modify the expression of fibronectin, CTGF, α-SMA, collagen I, collagen III, MMP2, MMP9, TGF-β, and p-Smad2/3 in the TGF-β/Smad signaling pathway. In summary, our findings suggest that PFD effectively counteracts the adverse effects of Ang II-induced CF proliferation and fibrosis, and modulates the TGF-β/Smad signaling pathway and *PARP9* expression. This identifies a potential therapeutic approach for managing myocardial fibrosis.

## Introduction

Cardiac arrhythmias, characterized by deviations in normal heart rhythm [1], have garnered significant attention given their association with increased morbidity and mortality [2]. Originating from disruptions in the cardiac electrical conduction pathway or aberrations in the myocardial action potential [3, 4], these arrhythmias can manifest in a plethora of ways, from benign palpitations to life-threatening ventricular fibrillation [5, 6]. In contrast, heart failure (HF) is a debilitating clinical condition that represents the inability of the heart to meet the metabolic needs of the body, primarily caused by pathologies, such as hypertension, coronary artery disease, and myocardial infarction [7]. A notable nexus between cardiac arrhythmias and HF has been discerned in contemporary research [8, 9]. Not only are HF patients more susceptible to arrhythmias, but sustained arrhythmias, in turn, can amplify HF severity by diminishing cardiac efficiency and fostering detrimental cardiac remodeling [10]. This bidirectional intricacy is further accentuated by common risk determinants, such as age, obesity, and diabetes [11]. Presently, while a myriad of treatments ranging from pharmacological interventions to device implantations exists for both conditions [12], the coexistence of arrhythmia and HF often complicates clinical management, making prognostication challenging [13].

Within the scope of the detrimental cardiac remodeling associated with cardiac arrhythmias, fibrotic processes have emerged as crucial culprits [14]. Cardiac fibrosis is characterized by excessive accumulation of extracellular matrix proteins in the interstitial cells of the heart, and in its etiology, angiotensin II (Ang II) is an important player [15]. The activation of cardiac fibroblasts (CFs) and their differentiation into myofibroblasts, highlighted by α-SMA, collagen I, and fibronectin expression, underscores this pathological remodeling [16]. Notably, Ang II not only fuels fibroblast proliferation and migration but also instigates the release of profibrotic factors, with transforming growth factor-beta 1 (TGF-β1) at its forefront [17]. This cytokine, acting through intracellular mediators, such as Smad2 and Smad3, further amplifies the fibrotic cascade [18]. Pirfenidone (PFD) is an antifibrotic drug that is widely used in the treatment of diseases associated with fibrosis in organs, such as the lungs, renal tubular mesenchyme, and liver [19–21]. Research suggests that PFD primarily mitigates pressure overload-induced cardiac fibrosis and dysfunction by preventing the signaling pathway of TGF-β1/Smad3 [22]. A Study by Fu et al. [23] also proposed a promising therapeutic strategy involving PFD-loaded nanodroplets combined with an acellular peritoneal matrix for alleviating myocardial fibrosis post-myocardial infarction in rats. Although the deleterious effects of Ang II and its downstream TGF-β signaling in cardiac fibrosis have been demonstrated, a comprehensive understanding of the regulators, such as PFD, that counteract this transition remains an uncharted frontier in cardiovascular research.

A member of the poly (ADP-ribose) polymerase (PARP) family, the *PARP9* gene is becoming more and more known for its involvement in a number of illnesses, mostly inflammatory and cancerous conditions [24–26]. According to recent studies, *PARP9* regulates immune cell activities and inflammatory responses [27, 28]. It may also control the activity of different immune cells and have an impact on the generation of inflammatory cytokines [29]. Additionally, recent studies suggested that *PARP9* affects immune function and may influence the response of the body to viral infection [30, 31]. Although these fields are not well researched, *PARP9* may be implicated in other disease processes since it is involved in DNA repair and cellular stress responses [32, 33]. In addition, since fibrotic and inflammatory processes are associated with the etiology and development of various heart diseases, the role of PARP9 in fibrosis and inflammation suggests it could be a promising therapeutic target for managing cardiac diseases. This hypothesis necessitates further research for confirmation.

Building on the established links between cardiac arrhythmias, HF, PFD, and the pivotal roles of TGF-β signaling and Ang II in cardiac fibrosis, to delve deeper into the molecular underpinnings that may serve as mitigators in this pathogenic cascade is still necessary. This study sets forth the intention of clarifying the function of *PARP9* in the setting of Ang II-induced cardiac fibrosis with or without PFD. By probing its potential implications and regulatory capacities, we aim to uncover whether *PARP9* overexpression can affect the fibrotic transformations instigated by Ang II with or without the addition of PFD. Unraveling this relationship not only deepens our understanding of the complex interplay at the molecular level but also paves the way for novel therapeutic strategies in managing the intertwined challenges of arrhythmias, and fibrosis.

## Materials and methods

### Data source and collection

The GSE42955 dataset was the main source of data used in the investigation. It was obtained from the public genomics data repository, Gene Expression Omnibus (GEO; https://www.ncbi.nlm.nih.gov/geo/). The dataset encompasses a total of 24 cardiac samples categorized as the case group and five samples as the control group. Within the case group, there are 12 samples from patients diagnosed with dilated cardiomyopathy (DCM) and another 12 samples from individuals with ischemic cardiomyopathy (ICM). In contrast, cardiac samples from people who do not appear to have any myocardial disease make up the control group.

### Weighted gene co-expression network analysis (WGCNA) on GSE42955 dataset

To elucidate intricate gene interactions and delineate gene modules strongly associated with ischemic cardiomyopathy (ICM), we employed WGCNA on the comprehensive GSE42955 dataset. Specifically, gene expression data covering the entire gene profile in the GSE42955 dataset was transformed into a scale-free network of co-expression. Applying the “WGCNA” program in R, we ensure strict adherence to the scale-free topology criterion, a hallmark of biologically meaningful networks. In this construct, each module contains a set of co-expressed genes, represented by different colors for clarity and identification. By aligning these gene modules with clinical phenotypes (case and control samples), we identified modules with significant associations with ICM.

### Differential expression and functional analysis

Utilizing the GEO2R tool, we analyzed the GSE42955 dataset to identify differentially expressed genes (DEGs). Our selection criteria for upregulated DEGs mandated a fold change (FC) exceeding 1.3, while downregulated DEGs required an FC below 0.77, both with a significance level of *P* < 0.05. Following DEG determination, the R VennDiagram tool was used to find the common genes between the yellow module and the GSE42955-downregulated DEGs. The Database for Annotation, Visualization, and Integrated Discovery (DAVID) database (http://david.abcc.ncifcrf.gov/) was then used to perform functional enrichment on these common genes. Extensive investigations using Gene Ontology (GO) and the Kyoto Encyclopedia of Genes and Genomes (KEGG) revealed related pathways and functions. Only results that had *P* values less than 0.05 were considered noteworthy.

### Protein–protein interaction (PPI) analysis and clinical diagnostic evaluation

Following the identification of the overlapping genes, we directed our analysis toward the proteins encoded by these 85 genes. A PPI network was constructed using Cytoscape software. Subsequently, the Cytohubba plugin within Cytoscape was employed, utilizing its molecular complex detection (MCODE), density of maximum neighborhood component (DMNC), and maximal clique centrality (MCC) algorithms to identify three pivotal network modules. To pinpoint key overlapping genes within these modules, an integrated analysis was performed using the “VennDiagram” package. Next, samples from the case and control groups of the GSE42955 dataset were evaluated to determine the expression levels of these overlapping genes. These key overlapping genes were subjected to a receiver operating characteristic (ROC) curve analysis using the R “timeROC” tool. The potential clinical diagnostic usefulness of the obtained results was evaluated by calculating area under the curve (AUC) values.

### Assessment of immune cell infiltration and correlation with *PARP9* via ImmuCellAI

An immune cell infiltration abundance tool called ImmuCellAI (http://bioinfo.life.hust.edu.cn/web/ImmuCellAI/) uses gene expression data to properly assess the relative abundance of several immune cell subtypes [34]. In this study, we used the ImmuCellAI program to analyze the infiltrating abundance of 24 immune cell subtypes in the GSE42955 dataset. The association between *PARP9* expression and the level of infiltration of various immune cell subsets was next investigated. The significance and strength of these connections were assessed using correlation coefficients and *P* values.

### Cell culture and treatment

CFs play a crucial role in heart development, function, and response to injury. In the present study, we utilized CFs induced by 1 µM of Ang II to emulate a pathological condition. The American Type Culture Collection (ATCC, American) was the initial source of the cells. Then, during varying lengths of time (24, 48, and 72 h), CFs were treated with PFD at various doses (0.5, 1.0, and 1.5 mg/mL). The cells were grown in DMEM supplemented with 10% fetal bovine serum (FBS) and 1% penicillin–streptomycin at 37 ^∘^C and 5% CO_2_ for the best possible growth and maintenance.

### Transfection assay

To probe the potential implications of *PARP9* in the context of Ang II-induced cardiac fibrosis, the CFs were subjected to transfection procedures. A *PARP9* overexpression plasmid (pcDNA3.1), along with a control vector, was utilized. Lipofectamine 2000 was used for transfections, and the manufacturer’s procedure was followed. Post-transfection, cells were incubated for a further 48 h before subsequent assays and analyses, ensuring sample time for optimal levels.

### Quantitative real-time-polymerase chain reaction (qRT-PCR) assay

Ang II-induced CFs were subjected to total RNA extraction utilizing the TRIzol Reagent. RNA purity and concentration were ascertained via spectrophotometric analysis. SuperScript III First-Strand Synthesis System assisted cDNA synthesis by reverse transcription of 1 g of the recovered RNA. Then, quantitative real-time PCR was performed using the Applied Biosystems 7500 Fast Real-Time PCR System, utilizing primers specific for *PARP9* (Forward: 5′- GAAATGTCCTGTGCCTCCAACT-3ʹ, Reverse: 5′- ACCTCATTGTCTATCTTCTCCACCTT-3ʹ), *α-SMA* (Forward: 5′-GGGACATCAAGGAGAAACTGTGT-3′, Reverse: 5′-TCTCTGGGCAGCGGAAAC-3′), *TGF-β* (Forward: 5′-TATGAGAGAATGTTGGTATG-3′, Reverse: 5′-CAATATCCTTCTGTTCCC-3′) as well as *GAPDH* (Forward: 5′-CGAGATCCCTCCAAAATCAA-3′, Reverse: 5′-TTCACACCCATGACGAACAT-3′), and the *GAPDH* as an internal reference. Computation of relative gene expression was executed employing the 2^-ΔΔCt^ methodology.

### Western blot (WB) assay

Protease inhibitor-supplemented RIPA buffer (1% Triton X-100, 1% sodium deoxycholate, 0.1% SDS, 0.15 M NaCl, 0.01 M sodium phosphate, pH 7.2) was used to produce cell lysates for protein analysis. A BCA protein assay kit (Pierce, Rockford, IL, USA) was used to measure the amounts of proteins. Comparable protein amounts were separated by SDS-PAGE and then put on PVDF membranes. Membranes were blocked and then treated with primary antibodies (1:1000 dilution) against PARP9, fibronectin, CTGF, α-SMA, collagen I, collagen III, MMP2, MMP9, TGF-β, Smad2, p-Smad2, Smad3, and p-Smad3 (all from Abcam, Cambridge, UK) overnight at 4 ^∘^C, then the relevant secondary antibodies (1:5000; Cell Signaling Technology, Danvers, MA, USA). The enhanced chemiluminescence (ECL) was used to view the signals.

### Cell counting kit-8 (CCK-8) assay

CFs stimulated with 1-µM Ang II, treated with different concentrations of PFD, or transfected with *PARP9* overexpression were cultured for 12 h. The CCK-8 test was used to measure cell viability at 24, 48, and 72 h following this incubation. A density of 5×10^3^ cells per well was used to seed cells in 96-well plates. Each well was given 10 µl of CCK-8 solution after the foregoing procedures, and it was incubated for two hours at 37 ^∘^C. The optical density (OD) at 450 nm was measured using a microplate reader to assess the viability of cell proliferation.

### Enzyme-linked immunosorbent assay (ELISA)

CFs were exposed to various PFD concentrations (0, 0.5, 1.0, and 1.5 mg/mL) during 48 h. The secretion of TGF-β in the culture supernatant of CFs, either treated or untreated with PFD, was quantified using commercial ELISA detection kits. The supplier of ELISA detection kits was R&D Systems in Minneapolis, Minnesota, USA. A microplate reader was used to detect absorbance at 450 nm after the proper processes of incubation and washing.

### Assay for cell migration

The migration potential of the CFs post-treatment was assessed using transwell chambers. In the migration test, cells were plated in serum-free media in the upper chamber and chemoattractant-supplied medium in the lower chamber containing 10% FBS. Non-migrated cells on the top surface were removed after incubation for 12 h. Rather, migrating cells on the bottom surface were labeled with DAPI, fixed with 4% paraformaldehyde, and counted under a microscope.

**Figure 1.**
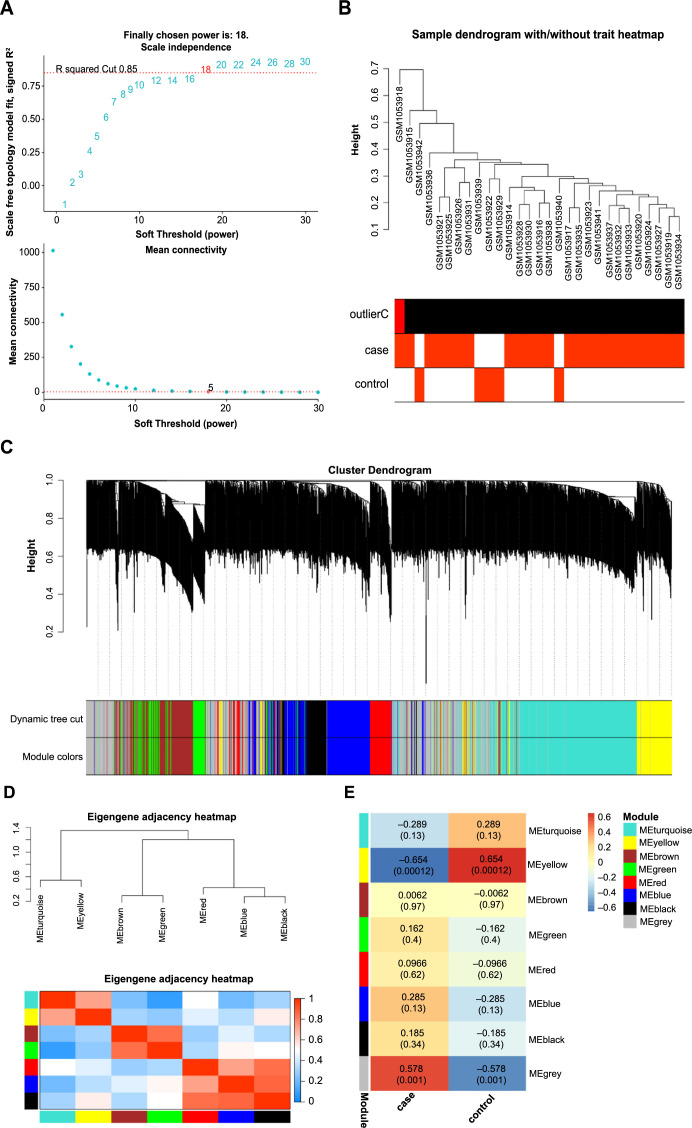
**Analysis of the GSE42955 dataset through WGCNA.** (A) Determination of the optimal soft-thresholding power, showcasing a value of 18 that ensures a scale-free topology model fit; (B) Sample dendrogram of the 29 samples from the GSE42955 dataset, illustrated with and without trait heatmap; (C) Clustering dendrogram showing the classification of genes into different modules according to their co-expression patterns, each module is represented by a different color; (D) Eigenegene adjacency heatmap, visualizing the relationship between the identified seven modules; (E) Correlation heatmap, correlation analysis between gene modules of different colors and two groups of samples of GSE42955. WGCNA: Weighted gene co-expression network analysis.

### Ethical statement

The data sourced from the public database are freely accessible, therefore, this study was not required to obtain authorization from a clinical ethics committee. The study adhered to the relevant regulations of the public database.

### Statistical analysis

Statistical analyses were performed using SPSS 25.0 software. Data from three independent biological experiments were presented as mean ± standard deviation (SD). Graphs were generated using GraphPad Prism 8.0. Student’s *t*-test was utilized for pairwise comparisons, with statistical significance set at *P* < 0.05. Each experiment was conducted in triplicate. R language packages were employed for statistical analyses, with differences between groups assessed using the student’s *t*-test, and significance determined at *P* < 0.05 for the mean.

## Results

### Identification of the yellow module as a key module in the GSE42955 dataset

As demonstrated in Figure 1A, the optimal soft-thresholding power, which ensures a scale-free topology model fit, was determined to be 18. Following the establishment of this soft-thresholding power, we proceeded to analyze the clustering of the 29 samples from the GSE42955 dataset (Figure 1B). Utilizing the WGCNA approach, based on their patterns of co-expression across the samples, genes were grouped into different modules, each of which was assigned a distinct color (Figure 1C). To decipher the interrelationships between these identified modules, eigengene adjacency was scrutinized (Figure 1D). Among the different modules, the correlation coefficient between the yellow module and the sample was 0.654, indicating a significant relationship (Figure 1E). This significant correlation underscored the potential biological relevance of the genes within the yellow module to the GSE42955 dataset.

### Enrichment analysis of 85 overlapping genes

**Figure 2.**
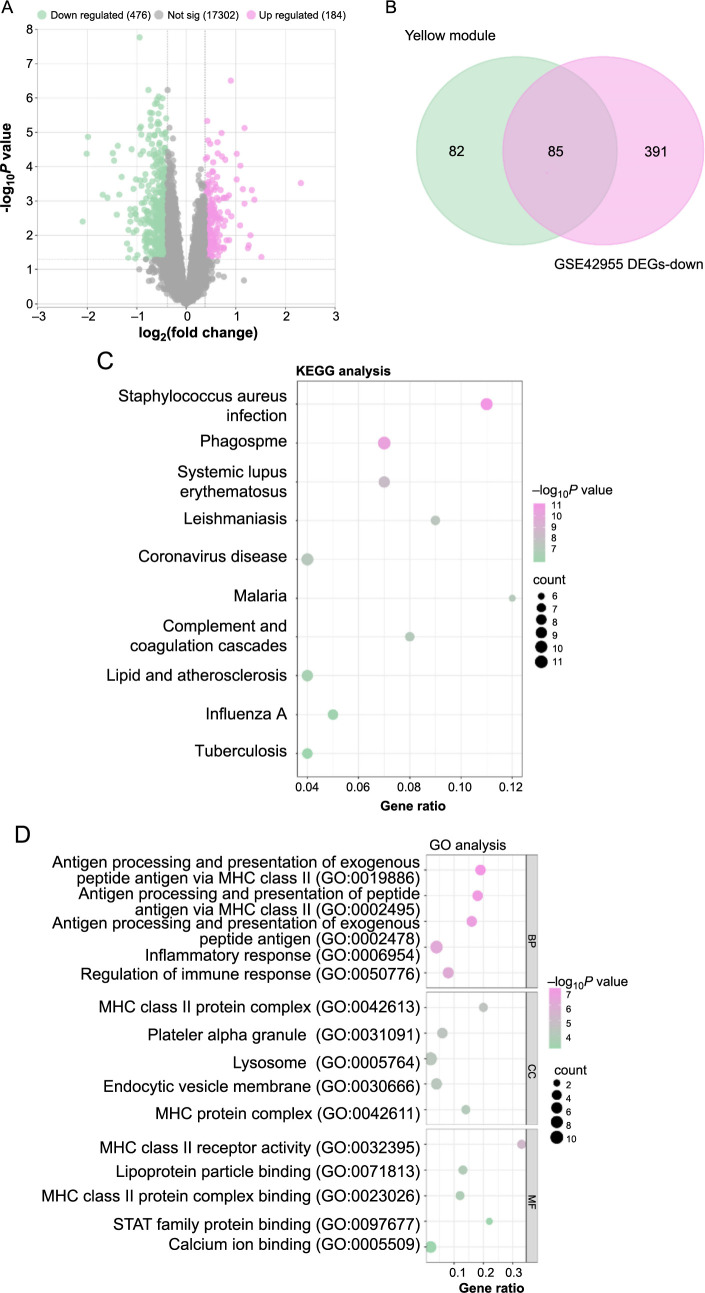
**Identification and enrichment analysis of overlapping genes in the GSE42955 dataset.** (A) Volcano map, the distribution of 660 DEGs in the GSE42955 dataset. Upregulated DEGs (184 in total) are shown in red, while downregulated DEGs (476 in total) are shown in green; (B) Venn diagram, overlap (middle) between genes of the yellow module (left) and downregulated DEGs in the GSE42955 dataset (right); (C) KEGG pathway enrichment study bubble graphic for 85 common genes. Each bubble represents a specific KEGG pathway. The *y*-axis displays the enriched pathways, the *x*-axis shows the gene ratio, and the size of the bubbles indicates how many enriched genes are present in each route; (D) Bubble plot of GO enrichment analysis of 85 overlapping genes covering BP, CC, and MF. The *x*-axis shows the percentage of genes, the *y*-axis displays the enriched GO terms, and the bubble size indicates how many genes are linked to each term. DEG: Differential gene expression; GO: Gene Ontology; KEGG: Kyoto Encyclopedia of Genes and Genomes; BP: Biological processes; MF: Molecular function.

From the GSE42955 dataset, we identified 660 DEGs, comprising 184 upregulated and 476 downregulated DEGs (Figure 2A). The subsequent analysis highlighted an intersection of 85 overlapping genes between the downregulated DEGs of the GSE42955 dataset and the yellow module containing 167 genes (Figure 2B). Through enrichment analysis, these genes were found to be predominantly associated with several KEGG pathways, including *Staphylococcus aureus* infection, Phagosome, Leishmaniasis, Malaria, and Influenza A (Figure 2C). In terms of GO annotations, the 85 genes were related to biological processes (BPs) like antigen processing and presentation of exogenous peptide antigen via MHC class II and inflammatory response, cell components (CCs), such as MHC class II protein complex and lysosome, and molecular function (MF) like MHC class II receptor activity and others (Figure 2D).

### Identification of *PARP9* as a key diagnostic biomarker through PPI and ROC curve analyses

**Figure 3.**
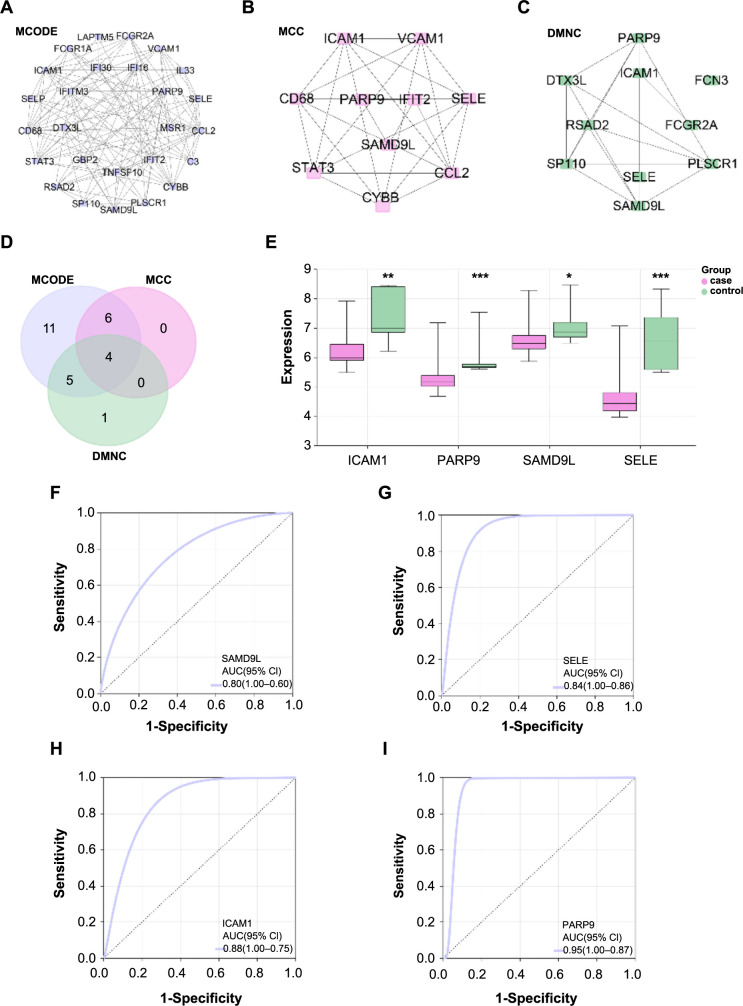
**PPI network analysis and validation of the candidate hub genes.** *P < 0.05, **P < 0.01, ***P < 0.001. (A) PPI network module derived from the MCODE algorithm, displaying 26 nodes and 120 edges; (B) PPI network module generated using the MCC algorithm, consisting of 10 nodes and 25 edges; (C) PPI network module constructed based on the DMMC algorithm, with 10 nodes and 16 edges; (D) Four important overlapping genes are shown in a Venn diagram that shows the intersection of the genes found in the three PPI network modules; (E) Box plot, expression levels of *ICAM1, PARP9, SAMD9L*, and *SELE* in GSE42955 samples, with the center line in each box representing the median; (F–I) ROC curve analysis for (F) *ICAM1*, (G) *PARP9*, (H) *SAMD9L*, and (I) *SELE*, respectively. False-positive rate (1-specificity) on the *x*-axis represents the percentage of negative samples that are incorrectly recognized as positive samples, while true-positive rate (sensitivity) on the *y*-axis represents the percentage of true positives that are accurately detected. PARP9: Polymerase 9; PPI: Protein–protein interaction; ROC: Receiver operating characteristic; MCODE: Molecular complex detection; MCC: Maximal clique centrality; AUC: Area under the curve.

After the identification of the 85 overlapping genes, a PPI network analysis was executed, leading to the derivation of three significant gene modules based on the MCODE (26 nodes and 120 edges), MCC (10 nodes and 25 edges), and DMMC (10 nodes and 16 edges) algorithms (Figures 3A–3C). From these modules, a set of four key overlapping genes emerged (Figure 3D). *ICAM1*, *PARP9*, *SAMD9L*, and *SELE* were mostly downregulated in the case group, according to further analysis of the expression levels of these genes within the GSE42955 samples, indicating their possible roles in the etiology or development of cardiac illness (Figure 3E). In the ROC curve analysis for these genes (Figures 3F–3I), *PARP9* exhibited the highest AUC value of 0.95, followed by *SELE* (AUC ═ 0.94), *ICAM1* (AUC ═ 0.88), and *SAMD9L* (AUC ═ 0.80). These high AUC values indicated the strong predictive capability and potential diagnostic significance of these genes, especially *PARP9*. Given its exceptional performance in the ROC analysis, *PARP9* was selected as the hub gene for further investigation in this study.

### *PARP9* expression correlates with infiltration of seven immune cell types

**Figure 4.**
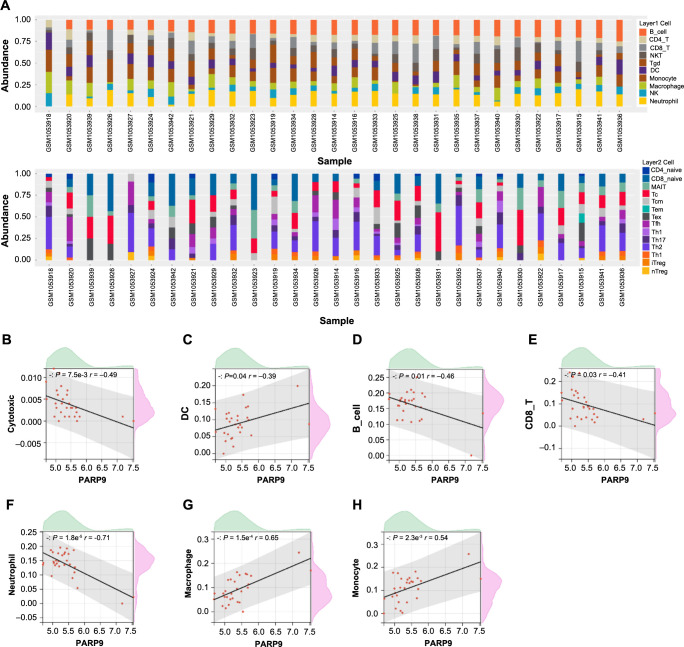
**Analysis of immune cell infiltration and its correlation with *PARP9* expression in GSE42955 samples.** (A) Infiltration abundance of 24 immune cell types across the 29 samples from the GSE42955 dataset. The *x*-axis denotes the individual samples, while the *y*-axis indicates the infiltration abundance. Different immune cell types are represented by different colors. (B–H) Scatterplots, correlations between *PARP9* expression and seven immune cell infiltrations, with the matching *P* value and Pearson correlation coefficient displayed in the top left corner for each. PARP9: Polymerase 9.

Utilizing the ImmuCellAI tool, the infiltration abundance of 24 distinct immune cell types was assessed in the GSE42955 samples. As illustrated in Figure 4A, a notably higher infiltration level was observed for immune cells, such as B cells, Neutrophils, CD8 T, Th2, and Tc. We investigated the connection between the previously described immune cells and *PARP9* expression. Remarkably, *PARP9* exhibited significant correlations with seven of these immune cell types. Specifically, *PARP9* demonstrated a significant negative correlation with cytotoxic (*r* ═ −0.49), B cell (*r* ═ −0.46), CD8_T (*r* ═ −0.41), and neutrophil (*r* ═ −0.71). On the contrary, significant positive correlations were noted between *PARP9* and DC (*r* ═ 0.39), macrophage (*r* ═ 0.65), and monocyte (*r* ═ 0.54) (Figures 4B–4H). These results imply that *PARP9* may be essential for regulating the immune microenvironment in the GSE42955 dataset samples.

### Effects of pirfenidone (PFD) on the proliferation activity, myofibroblast differentiation, and TGF-β expression of cardiac fibroblasts (CFs)

**Figure 5.**
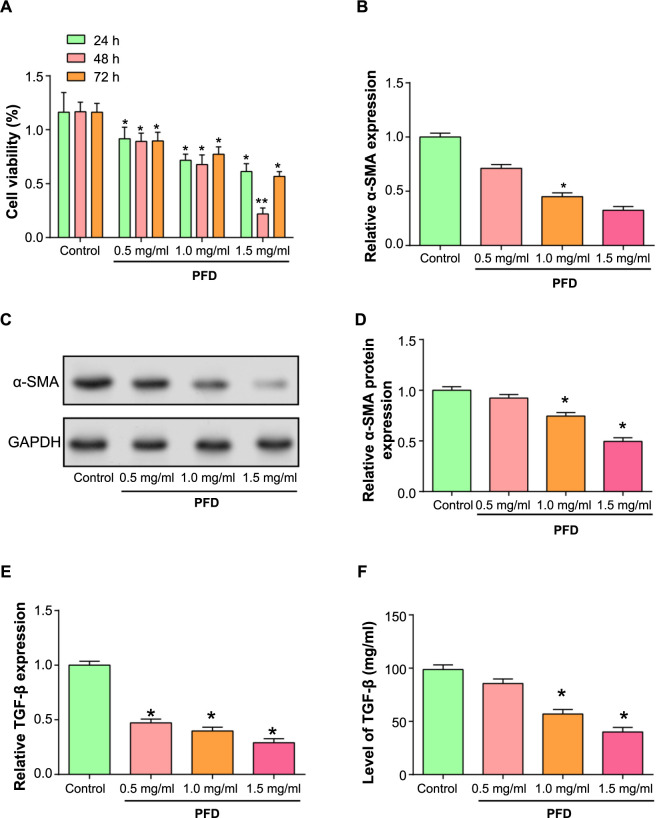
**Effects of PFD on CF proliferation and *TGF-β* expression.** *P < 0.05, **P < 0.01. (A) CCK-8 assay to examine the impact of different PFD concentrations on CF vitality over specified time intervals. The proportion of viable cells is shown on the *y*-axis, while various concentrations (0, 0.5, 1.0, and 1.5 mg/mL) during specified time intervals are represented on the *x*-axis; (B–D) qRT-PCR (B) and WB (C) analysis evaluating *α-SMA* mRNA expression levels and protein expression levels, respectively, in CFs treated with different concentrations of PFD. Visualization using Image J (D); (E) qRT-PCR to detect mRNA expression levels of *TGF-β* in CFs treated with different concentrations of PFD (F) ELISA to quantify TGF-β secretion in the cell culture supernatant. The *x*-axis represents different concentrations (0, 0.5, 1.0, 1.5 mg/mL), and the *y*-axis displays the concentration of TGF-β. CF: Cardiac fibroblast; PFD: Pirfenidone; qRT-PCR: Quantitative real-time polymerase chain reaction; WB: Western blot; CCK-8: Cell Counting Kit-8; ELISA: Enzyme-linked immunosorbent assay.

In this investigation, we assessed the impact of various PFD concentrations on the viability of CFs at various time intervals using the CCK-8 test (Figure 5A). Notably, treatment with 1.5-mg/mL PFD for 48 h showed the most significant inhibition of cell viability, prompting the selection of this condition for further studies. A sign of myofibroblast differentiation is the expression and organization of *α-SMA*. We used qRT-PCR (Figure 5B) and WB (Figures 5C and 5D) assays to assess the expression levels of *α-SMA* in CFs treated with different concentrations of PFD. The results showed significant downregulation at 1.0 and 1.5 mg/mL. At the mRNA level, studies of *TGF-β* expression in CFs under different concentrations of PFD revealed that *TGF-β* transcription was significantly reduced after exposure to PFD, as shown in Figure 5E. This finding was further validated through ELISA assay of TGF-β secretion in the cell culture supernatant (Figure 5F), supporting the conclusion that PFD influenced CF proliferation and *TGF-β* expression.

### PFD inhibits Ang II-induced proliferation and migration of CFs

**Figure 6.**
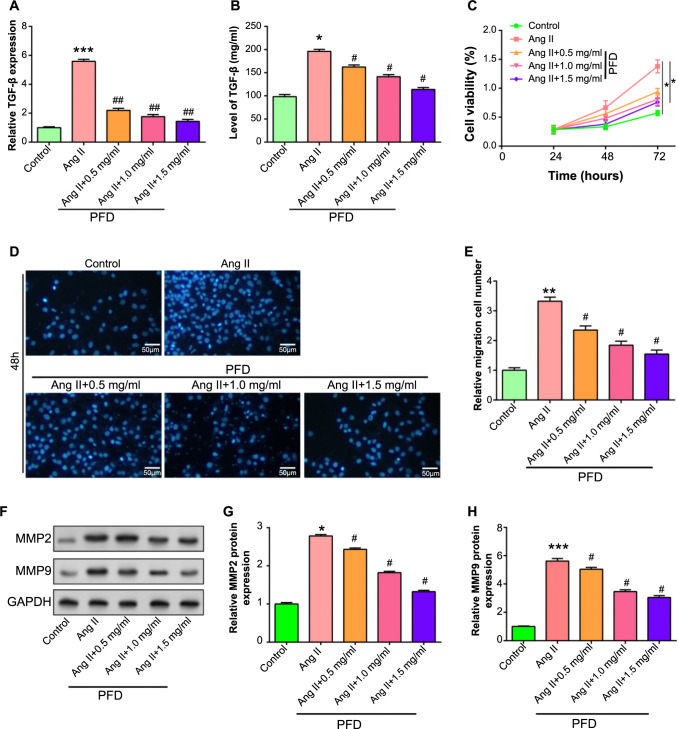
**Modulation of fibroblast response to Ang II and PFD treatment.** *P < 0.05, **P < 0.01, ***P < 0.001, ^#^*P* < 0.01. (A and B) qRT-PCR (A) and ELISA (B) analyses evaluating the TGF-β expression levels in CFs given varying PFD doses (0.5, 1.0, and 1.5 mg/mL) with or without Ang II stimulation. The *x*-axis represents the presence or absence of Ang II and the addition of varying PFD concentrations, while the *y*-axis represents the relative expression levels of *TGF-β* or the concentration of TGF-β. (C) Evaluation of cell proliferation through the CCK-8 assay. The graph depicts absorbance values representing cell proliferation under different treatment conditions. Time is shown by the *x*-axis, while cell viability is represented by the *y*-axis. (D and E) Transwell assay for cell migration. (D) Represents migrated cells, and (E) displays the quantification of migrated cells under different treatment conditions, assessed through the Tranwell assay. (F-H) WB analysis of migration-related proteins MMP2 and MMP9 in Ang II-stimulated CFs treated or untreated with PFD. (G) shows quantitative assessment of MMP2 expression using Image J, and (H) displays quantitative assessment of MMP9 expression utilizing Image J. CF: Cardiac fibroblast; Ang II: Angiotensin II; PFD: Pirfenidone; qRT-PCR: Quantitative real-time polymerase chain reaction; WB: Western blot; CCK-8: Cell Counting Kit-8; ELISA: Enzyme-linked immunosorbent assay.

*TGF-β* expression levels were significantly upregulated when 1-µM Ang II was used to stimulate CFs. It is significant that the elevation of *TGF-β* expression brought on by Ang II was successfully suppressed by concurrently treating cells with several doses of PFD (0.5, 1.0, and 1.5 mg/mL) for 48 h (Figure 6A). This inhibitory effect was further confirmed by subsequent experimental validation by ELISA (Figure 6B). The CCK-8 and transwell assays were then used to measure cell migration and proliferation (Figures 6C–6E). The Ang II group exhibited a considerable increase in both cell proliferation activity and migration number as compared to the control group. However, there was a dose-dependent reduction in cell migration number and proliferation activity when Ang II was coupled with various PFD doses. These findings suggested that PFD reduced the proliferation and migratory reactions in CFs caused by Ang II. WB experiments then evaluated the levels of migration-related proteins and extracellular matrix synthesis-related proteins, especially MMP2 and MMP9 (Figures 6F–6H). After Ang II induction, MMP2 and MMP9 protein levels significantly increased in CFs. Interestingly, PFD administration was found to inhibit the impacts of Ang II on MMP2 and MMP9 expression. This observation suggested a potential role for PFD in regulating matrix remodeling and migration of Ang II-stimulated CFs.

### PFD inhibits Ang II-induced CF fibrosis and downregulates *PARP9* expression

**Figure 7.**
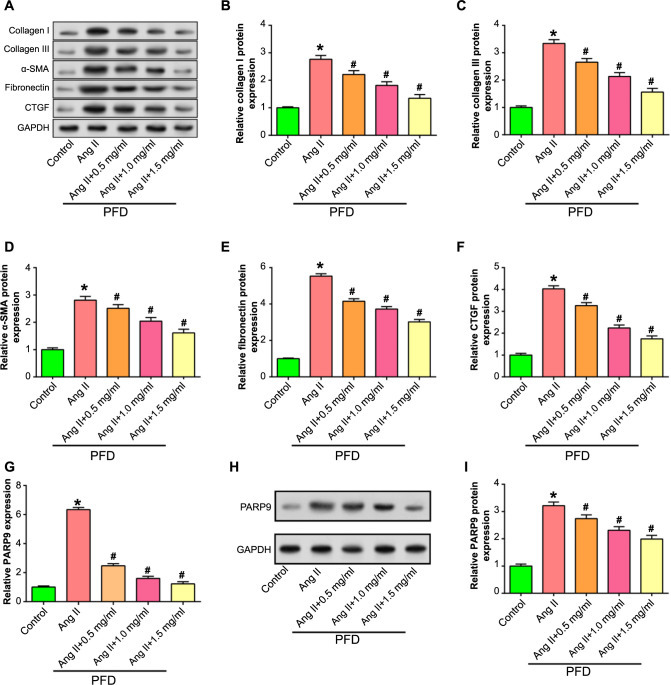
**Expression analysis of fibrosis markers and *PARP9* in CFs under Ang II and PFD treatment.** *P < 0.05, ^#^*P* < 0.05. (A–G) WB analysis assessing and visualizing the level of collagen I (B), collagen III (C), α-SMA (D), fibronectin (E), and CTGF (F) in CFs handled with varying concentrations of PFD in the presence or absence of Ang II. The *x*-axis represents the treatment conditions (Control, Ang II, Ang II + 0.5 mg/mL PFD, Ang II + 1.0 mg/mL PFD, Ang II + 1.5 mg/mL PFD), and the *y*-axis represents the relative protein expression levels. (H and I) qRT-PCR and WB analysis evaluating the expression of *PARP9* in CFs treated with varying concentrations of PFD if Ang II is present or not. The *x*-axis represents the treatment conditions (Control, Ang II, Ang II + 0.5 mg/mL PFD, Ang II + 1.0 mg/mL PFD, Ang II + 1.5 mg/mL PFD), and the *y*-axis represents the relative expression levels of *PARP9*. CF: Cardiac fibroblast; PARP9: Polymerase 9; Ang II: Angiotensin II; PFD: Pirfenidone; qRT-PCR: Quantitative real-time polymerase chain reaction; WB: Western blot.

To evaluate the degree of cellular fibrosis, WB assay was used to detect the level of α-SMA, collagen I, collagen III, CTGF, and fibronectin in CFs after Ang II induction and treatment with different concentrations of PFD. As shown in Figures 7A–7F, compared with the control group, the expression of α-SMA, collagen I, collagen III, fibronectin, and CTGF increased notably in the Ang II group. However, when comparing the Ang II group to the groups treated with 0.5, 1.0, and 1.5-mg/mL PFD, there was a substantial decrease in protein expression. This decreasing trend was most evident in the Ang II + 1.5-mg/mL PFD group. Subsequently, we evaluated the expression of *PARP9* in CFs treated with different concentrations of PFD under Ang II induction. When comparing the Ang II group to the control group, the results revealed a substantial increase in *PARP9* expression. As opposed to Ang II induction alone, PARP9 expression was dose-dependently decreased following treatment with Ang II in conjunction with 0.5, 1.0, and 1.5-mg/mL PFD (Figures 7G–7I). These findings collectively indicate that PFD inhibits Ang II-induced CF fibrosis and downregulates *PARP9* expression, with the PFD concentration being most significant at 1.5 mg/mL, so this concentration was selected for subsequent experiments.

### *PARP9* overexpression reverses the inhibitory effects of PFD on Ang II-induced cell proliferation, migration, and fibrosis

**Figure 8.**
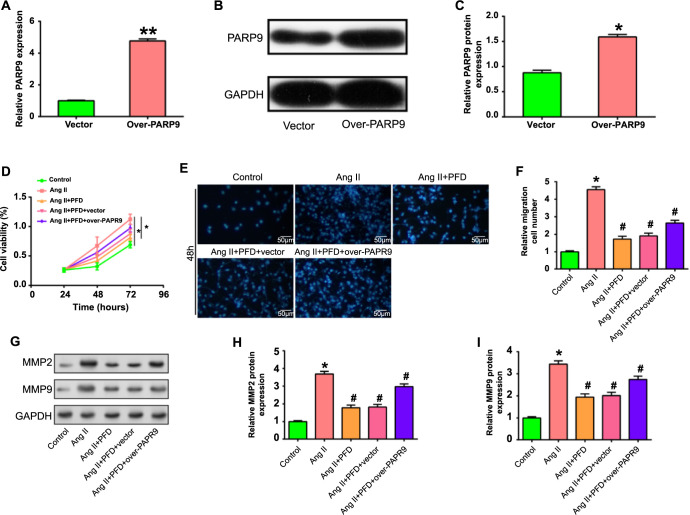
**Effects of *PARP9* overexpression and PFD treatment on Ang II-induced proliferation, migration and fibrosis of CFs.** *P < 0.05, **P < 0.01, ^#^*P* < 0.05. (A–C) qRT-PCR (A) and WB analysis (B and C) illustrating the successful upregulation of *PARP9* in CFs following overexpression. The treatment conditions (control, *PARP9* overexpression) are shown by the *x*-axis, while the relative expression levels of *PARP9* are represented by the *y*-axis. (D) The CCK-8 assay was employed to depict the proliferative activity of CFs induced by Ang II under different conditions. The *x*-axis represents time, and the *y*-axis represents cell viability. (E and F) Transwell experiments evaluating the migration of Ang II-induced CFs under different conditions. (E) Represents migrated cells, and (F) shows quantitative analysis of migrated cells. The treatment circumstances are shown on the *x*-axis, and the quantity of migrating cells is shown on the *y*-axis. (G–I) WB analysis assessing the protein expression levels of MMP2 and MMP9 in Ang II-induced CFs under various conditions. (H) Represents MMP2 expression quantified using Image J, (I) shows MMP9 expression quantified using Image J. The treatment conditions are shown by the *x*-axis, while the relative expression levels of MMP2 and MMP9 are represented by the *y*-axis. CF: Cardiac fibroblast; PARP9: Polymerase 9; Ang II: Angiotensin II; PFD: Pirfenidone; qRT-PCR: Quantitative real-time polymerase chain reaction; WB: Western blot; CCK-8: Cell Counting Kit-8.

qRT-PCR analyzed the significant overexpression efficiency of *PARP9* in CFs, and WB experiments further confirmed this observation at the protein level (Figures 8A and 8B). CCK-8 assay found that the proliferative activity of CFs induced by Ang II was greatly elevated. However, with the introduction of PFD (1.5 mg/mL), the promoting effect on cell proliferation was significantly inhibited. Notably, PARP overexpression had a synergistic protective effect when Ang II and PFD were treated simultaneously. Although the inhibition of proliferation was reduced relative to Ang II induction alone, proliferation rates were still elevated compared with controls (Figure 8D). Similarly, transwell experiments confirmed the CCK-8 assay results, indicating that *PARP9* overexpression regulates the migration of CFs induced by Ang II combined with PFD (Figures 8E and 8F). In addition, WB analysis also evaluated the protein expression levels of MMP2 and MMP9 under different conditions. The results showed that *PARP9* overexpression could reverse the PFD-induced suppression of MMP2 and MMP9 expression to some extent in the presence of Ang II (Figures 8G–8I). Taken together, these results indicated a complex interplay between *PARP9*, Ang II, and PFD in CF cellular processes.

**Figure 9.**
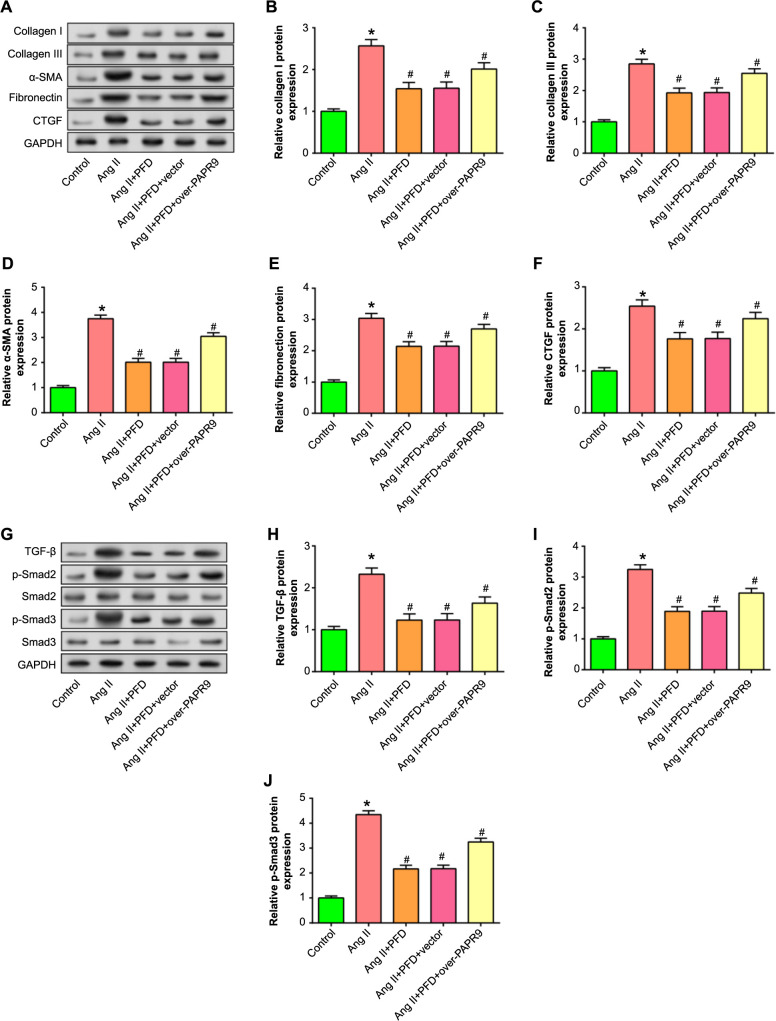
**Regulation of CF fibrosis and TGF-β/Smad signaling axis by *PARP9* overexpression and PFD.** *P < 0.05, ^#^*P* < 0.05. (A–F) WB analysis assessing the amounts of collagen I (B), collagen III (C), α-SMA (D), fibronectin (E), and CTGF (F) protein expression in CFs under different conditions. The *x*-axis represents the treatment conditions (Control, Ang II, Ang II + PFD, Ang II + PFD + *PARP9* Overexpression), and the relative expression levels of fibrotic markers are shown on the *y*-axis. (G–J) WB analysis assessing and visualizing TGF-β (H), p-Smad2 (I), Smad2, p-Smad3 (J), and Smad3 protein expression levels in CFs under different conditions. The conditions include control, Ang II induction, Ang II and PFD combined therapy, and Ang II, PFD, and *PARP9* overexpression combined treatment. CF: Cardiac fibroblast; PARP9: Polymerase 9; Ang II: Angiotensin II; PFD: Pirfenidone; WB: Western blot.

### Effects of *PARP9* overexpression and PFD on fibrosis markers and TGF-β signaling in Ang II-stimulated CFs

To gain further insights into the impact of *PARP9* on CF fibrosis, WB analysis was employed. In comparison to the control group, induction by Ang II resulted in elevated protein expression levels of α-SMA, collagen I, collagen III, CTGF, and fibronectin. However, the addition of PFD effectively inhibited this induction. Moreover, the inhibitory effect was reversed upon the subsequent introduction of *PARP9* overexpression (Figures 9A–9F). *TGF-β* is a critical cytokine that induces fibroblast activation and differentiation, thereby promoting fibrogenesis [35]. Smad2 and Smad3 are intracellular mediators of the TGF-β signaling pathway [36]. Further delving into the signaling cascade, TGF-β and its downstream effectors, Smad2, p-Smad2, Smad3, and p-Smad3, are paramount in elucidating fibrotic pathways and the ensuing fibroblast activation [37]. The expression of the TGF-β/Smad signaling pathway was evaluated by WB analysis. Following Ang II induction, CFs exhibited a noticeable increase in protein levels of p-Smad2, p-Smad3, and TGF-β compared to the control group. The addition of PFD effectively suppressed this elevation. Furthermore, the introduction of *PARP9* overexpression reversed this suppression (Figures 9G–9J). These results emphasized the regulating effects of PFD and overexpression of *PARP9* on the TGF-β/Smad signaling pathway and Ang II-stimulated CF fibrosis.

## Discussion

Within this research, we delved deeper into the molecular intricacies associated with cardiac fibrosis, particularly focusing on the critical function of PARP9 in the framework of fibrotic pathways generated by Ang II. The prominence of the yellow module in the GSE42955 dataset and its substantial correlation provided a foundation to explore the potential regulatory genes that regulate the course or pathophysiology of cardiac disease. The identification of *PARP9*, among other genes, stood out not only for its diagnostic significance but also for its potential therapeutic implications in cardiac fibrosis [38, 39]. Our findings shed light on how *PARP9* can function as a moderator in the complex interplay between Ang II-induced proliferative activities, fibrosis marker elevation, and TGF-β signaling pathway activation. Furthermore, *PARP9* has not only regulated immune cell infiltration but has also strategically controlled the migration and proliferation of CFs after Ang II induction. New insights into cardiac fibrosis emerged from the notable connection between *PARP9*, immunological regulation, and fibrotic transformation. This implies broader implications for the understanding of heart disease development and progression.

By conducting an in-depth bioinformatics analysis of the GSE42955 dataset, we discerned 85 overlapping genes between its yellow module and the downregulated DEGs. Remarkably, these genes manifested significant associations with multiple KEGG pathways, most prominently with *Staphylococcus aureus* infection and the phagosome pathway. Such pathways have historically been interlinked with immunomodulatory roles across diverse cardiac ailments. For instance, *Staphylococcus aureus* infections are known to provoke systemic inflammatory responses, potentially exacerbating myocardial injury and subsequent dysfunction [40]. In a similar vein, the role of the phagosome pathway, especially in orchestrating cardiac immune responses post-ischemic events, underscores its pertinence to our findings [41]. Beyond this, the elucidated GO annotations spotlight key BPs, suggesting a nuanced interplay between immune mechanisms and cardiac fibrotic events. A case in point is the dysregulation of MHC class II presentation, which has been linked to autoimmune manifestations in conditions like myocarditis [42]. In summation, these insights hint that the identified genes, alongside their intertwined pathways, might play a pivotal role in bridging immune dynamics with the evolution of cardiac pathologies.

Following the elucidation of co-expression modules and the identification of 85 intersecting genes, our analysis took a deeper dive into the intricate PPI network. This meticulous exploration identified three pivotal gene modules, leading to the spotlight on four overlapping genes: *ICAM1*, *PARP9*, *SAMD9L*, and *SELE*. When contextualized with the GSE42955 samples, there was a marked downregulation of these genes in the case group, with *PARP9* especially drawing attention due to its pronounced downregulation and its robust AUC value of 0.95 in the ROC analysis. Part of the PARP family, *PARP9* is involved in DNA damage repair, gene transcription, and cellular stress response [43]. The study by Szántó M. et al. pointed to the association of *PARP9* with lipid metabolism and liver function [44]. Interestingly, a study by Iwata H. et al., which revealed a link between the *PARP9–*PARP14 axis and coronary artery disease in humans, highlighted the role of *PARP9* in regulating macrophage activation [45]. These findings further emphasize the importance of *PARP9* in the cardiac domain. Utilizing the ImmuCellAI tool, we delved into the immune cell infiltration patterns in the GSE42955 samples. Notably, heightened infiltration levels were observed for B cells, neutrophils, and CD8_T cells. The expression of *PARP9* manifested negative correlations with cytotoxic and neutrophil cells, but positive correlations with DC, macrophage, and monocyte cells. Lymphocytes, including T and B cells, have been linked to post-injury cardiac remodeling and autoimmune disorders like myocarditis [46]. Such intricate interplays between immune cells and cardiomyocytes significantly influenced the trajectory of various cardiac conditions. This multifaceted relationship underscores the central role of *PARP9* in shaping the immune microenvironment. In light of this, exploring immune cell-based interventions opens new horizons for heart disease management.

PFD, recognized as a broad-spectrum antifibrotic agent, has been extensively investigated in various studies. Avila et al. [47] highlighted its promising therapeutic role in cardiac diseases, particularly in diabetic cardiomyopathy, addressing structural concerns such as fibrosis and stiffness. A study by Lopez-de la Mora DA et al. emphasized the potential of PFD to alleviate scar tissue deposition by reducing key fibrotic markers like TGF-β1, TNF-α, PDGF, and COL1A1, underscoring its positive effects on inflammation and fibrogenesis [48]. Li et al. [49] further demonstrated the cardiac protective effects of PFD by attenuating fibrosis via the AT1R/p38 MAPK/RAS pathway and activating liver X receptor-α (LXR-α) after myocardial infarction. In our subsequent in vitro experiments, PFD exhibited dose-dependent inhibition of CF proliferation, and it suppressed α-SMA and TGF-β expression, hindering myofibroblast differentiation. Notably, PFD hindered Ang II-caused CF migration, proliferation, and fibrosis while downregulating *PARP9* expression. According to Zhang et al. [50] PFD may have inhibitory impacts on fibroblast adhesion, migration, and proliferation that are mediated by the PI3K/AKT signaling pathway. Moreover, local application of PFD effectively reduced epidural fibrosis induced by laminectomy, indicating its potential as a safe and effective intervention. Hall et al. [51] also found that PFD exhibits potential as an antifibrotic agent in dermal fibrosis by inhibiting TGF-β1-induced myofibroblast differentiation, cell proliferation, and migration. The research of Vu et al. [52] indicated that inhaled interferon-gamma (IFN-γ) and PFD demonstrate distinct and complementary antifibrotic effects on normal and idiopathic pulmonary fibrosis (IPF) lung fibroblasts. The combination of these agents showed potential synergistic or additive effects, inhibiting fibroblast proliferation, migration, and differentiation induced by TGF-β1 and PDGF-BB. These findings collectively suggested the versatility of PFD as an agent of therapy with the potential for combination therapies targeting fibrotic disorders in different organ systems.

In further in vitro experiments, our findings emphasized that Ang II induction led to a decrease in *PARP9* mRNA and protein levels in CFs. Interestingly, while PFD attenuated the proliferative activity of CFs produced by Ang II, overexpression of *PARP9* reversed the detrimental impact of PFD on Ang II-induced cell proliferation, migration, and fibrosis. This discovery underscored the ability of PARP9 overexpression to counteract the proliferative impact of PFD in the context of Ang II induction. Delving deeper into the regulatory role of *PARP9*, its overexpression also mitigated the inhibiting impact of PFD on key fibrotic markers induced by Ang II, namely, collagen I, collagen III, α-SMA, CTGF, and fibronectin. In addition, the TGF-β1 signaling pathway stands out as a pivotal player in this fibrotic narrative. TGF-β, a potent cytokine, is intricately involved in diverse cellular activities, including fostering fibrosis via driving the transition of fibroblasts to myofibroblasts [53]. The downstream effectors of this pathway, p-Smad2 and p-Smad3, are intracellular mediators essential to this transformation process [54]. In our study, these proteins exhibited a significant increase after Ang II induction, which was markedly modulated by PFD. Notably, *PARP9* overexpression reversed the protein level reduction induced by the addition of PFD. These results illuminated the intricate interplay between PFD, *PARP9*, and the TGF-β/Smad signaling pathway in modulating CF behavior and fibrotic responses.

## Conclusion

Our comprehensive study elucidated the significant role of *PARP9* in CF function and fibrotic remodeling, particularly in the context of Ang II induction. We observed the efficacy of PFD in mitigating CF proliferation, migration, and fibrosis, and notably, its influence on TGF-β expression. The mitigative effect of *PARP9* overexpression on pivotal fibrosis markers, such as α-SMA, collagen 1, and fibronectin, further underscores its regulatory significance. Central to our findings is the identification of the TGF-β signaling pathway, and its downstream effectors, p-Smad2 and p-Smad3, as being paramount in fibrotic regulation. Additionally, the inhibitory effects of PFD on Ang II-induced fibroblast activation, migration, and fibrosis were reversed by *PARP9* overexpression, further emphasizing the regulatory role of *PARP9* in CF behavior. These insights collectively highlighted the potential therapeutic value of targeting *PARP9*, particularly in cardiac conditions exacerbated by fibrotic remodeling. However, there are some limitations in our study, such as the absence of data displaying the extent and distribution of cellular fibrosis by immunofluorescence microscopy or electron microscopy, and the absence of corresponding histological examinations. Future research could further explore the mechanistic intricacies and potential applications of *PARP9* modulation in arrhythmia-associated disorders.

## Data Availability

The datasets used and/or analyzed during the current study are available from the corresponding author upon reasonable request.
